# A Selective Biogeography-Based Optimizer Considering Resource Allocation for Large-Scale Global Optimization

**DOI:** 10.1155/2019/1240162

**Published:** 2019-07-10

**Authors:** Meiji Cui, Li Li, Miaojing Shi

**Affiliations:** ^1^College of Electronics and Information Engineering, Tongji University, Shanghai 201804, China; ^2^Shanghai Institute of Intelligent Science and Technology, Tongji University, Shanghai 201804, China; ^3^Inria, Univ Rennes, CNRS, IRISA, 35000 Rennes, France

## Abstract

Biogeography-based optimization (BBO), a recent proposed metaheuristic algorithm, has been successfully applied to many optimization problems due to its simplicity and efficiency. However, BBO is sensitive to the curse of dimensionality; its performance degrades rapidly as the dimensionality of the search space increases. In this paper, a selective migration operator is proposed to scale up the performance of BBO and we name it selective BBO (SBBO). The differential migration operator is selected heuristically to explore the global area as far as possible whist the normal distributed migration operator is chosen to exploit the local area. By the means of heuristic selection, an appropriate migration operator can be used to search the global optimum efficiently. Moreover, the strategy of cooperative coevolution (CC) is adopted to solve large-scale global optimization problems (LSOPs). To deal with subgroup imbalance contribution to the whole solution in the context of CC, a more efficient computing resource allocation is proposed. Extensive experiments are conducted on the CEC 2010 benchmark suite for large-scale global optimization, and the results show the effectiveness and efficiency of SBBO compared with BBO variants and other representative algorithms for LSOPs. Also, the results confirm that the proposed computing resource allocation is vital to the large-scale optimization within the limited computation budget.

## 1. Introduction

Evolutionary algorithms (EAs) are efficient tools to solve complex optimization problems. Biogeography-based optimization (BBO) [1], proposed by Simon in 2008, is inspired by biogeography regarding the migration of species between different habitats, as well as the evolution and extinction of species. Assuming an optimization problem and some candidate solutions, each habitat represents a candidate solution, the suitability of the habitat is the fitness of the optimization problem, and the habitat features represent decision variables. According to the biogeography theory, a superior solution tends to share more promising information with the inferior one by the way of migration, specifically high emigration as well as low immigration in this case, and vice visa. Also, mutation may occur with certain probability in accordance with the biogeography evolution.

As a new yet promising EA, BBO has been applied to solve single-objective problems [2], multiobjective problems [3, 4], and constrained problems [5] to some success. What's more, some extensions of BBO have been proposed to improve its performance [6, 7]. BBO has been extensively explored to deal with many real-word complex problems, such as manufacturing system scheduling [8], supply chain design optimization [9], and hub competitive location [10]. However, it has been reported that the performance of BBO degraded rapidly when the problem dimension increases [11]. With the advent of big data era, the scalability of an EA is a significant indicator to be considered.

In comparison with traditional optimization problems, modern optimization problems [12, 13] tend to involve a large number of decision variables, which is also conceptualized as large-scale optimization problems (LSOPs). Owing to the explosion of search space and interdependencies among decision variables, LSOPs cannot be tackled in reasonable time by conventional EAs. This has made LSOPs an open and challenging problem, which has attracted intensive attention in recent trends.

Existing methods to deal with LSOPs can be divided into two categories, i.e., decomposition methods and nondecomposition methods. Nondecomposition methods refer to those exploring some special operators [14], local search [15], and hybrid algorithms [16], etc. to improve the search ability of conventional EAs. While decomposition methods, also known as divide and conquer (DC), take advantages of the modularity characteristic of optimization problems and divide the high-dimensional problem into several low-dimensional subproblems. These subproblems can thus be evolved with a certain EA independently in a more efficient manner. Due to the dimensionality mismatch brought by DC, which implies that the subsolution cannot be evaluated by the original objective function directly, it is a natural way to complement the subsolution to be evaluated as a complete solution by the combination of the representative of each subproblem, also known as cooperative coevolution (CC).

Compared with nondecomposition methods, the DC framework is more efficient and therefore more popular. Recent works along this line mainly focus on the grouping strategy for subproblem division, e.g., random grouping [17] and recursive differential grouping [18]; on the other hand, the performance of optimizers and the allocation of computing resources among subproblems within limited computational budget are also crucial but have not been largely explored yet. Therefore, it is meaningful to investigate new algorithms for LSOPs with the aim of making a new attempt for this difficult problem as well as exploring extensions of BBO.

In this paper, we intend to scale up the performance of BBO to solve the LSOPs. We propose a novel Selective Migration Operator (SMO) to balance exploration and exploitation. If the selected emigration individual is better than the immigration one, once the migration occurs, a differential migration operator with a relatively large value is chosen to share more good information with the poor individual; otherwise, a normal distributed random value with small variance is applied for local search. Through the selective migration operator, a more rapid and efficient search process can be conducted in reasonable time. Furthermore, the DC framework is adopted to enhance the ability to solve high-dimensional problems. To solve the problem of subgroup contribution imbalance in the context of DC, a simple and efficient computing resource allocation strategy is proposed in the end.

The paper is set as follows. In Section 2, the BBO algorithm and Large-Scale Optimization (LSO) are briefly introduced.  Section 3 presents our Selective Biogeography-Based Optimization (SBBO) with selective migration operator and a more efficient computing resource allocation strategy for DC framework.  Section 4 depicts the experiments and corresponding results, followed by some analysis. Finally, conclusion and future work are drawn in Section 5.

## 2. Background

### 2.1. Biogeography-Based Optimization

In biogeography, there are two important terms, namely, habitat suitability index (HSI) and suitability index variables (SIVs) [1]. HSI is used to evaluate the living environment for each habitat while SIVs are the influencing factors of HSI. For an optimization problem, the population, i.e., habitats, represents a set of candidate solutions, while the SIVs of habitats are considered as the feature representations of the candidate solutions. Therefore, the evolutionary algorithm inspired by biogeography, i.e., biogeography-based optimization, is naturally used to solve different kinds of optimization problems.

**Figure 1:**
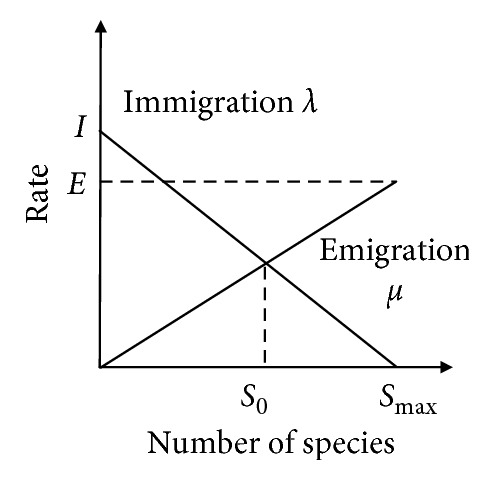
Species migration model of an island.

There are two main operators in canonical BBO, i.e., migration operator and mutation operator. The migration operator is to share search information among individuals, and the mutation operator is to enhance the population diversity. The immigration rate *λ*_*i*_ and emigration rate *μ*_*i*_ of a habitat *H*_*i*_ can be calculated by the migration model, which is shown in Figure 1 [1]. More specifically, we adopt a simplified linear migration model to demonstrate the process, where the migration model is the function of the number of species. When the number of species increases, fewer species can survive for immigration and more species tend to emigrate to other habitats, and vice visa. The corresponding immigration and emigration rates are given by

(1) $$\begin{array}{c} \lambda_{i}=I\left(1-\frac{S_{i}}{S_{\max}}\right), \end{array}$$

(2) $$\begin{array}{c} \mu_{i}=E\left(\frac{S_{i}}{S_{\max}}\right), \end{array}$$

where *I* is the maximum immigration rate, *E* is the maximum emigration rate, *S*_*i*_ is the number of species of the habitat *H*_*i*_, and *S*_max_ is the maximum number of species. In BBO, the habitat with more species signifies a better solution. That being said, a better solution has lower immigration rate and higher emigration rate, so that it can share promising information with other solutions and is less likely to be destroyed due to migration.

Next, the migration can be expressed as

(3) $$\begin{array}{c} H_{i}\left(\text{SIV}\right)⟵H_{j}\left(\text{SIV}\right), \end{array}$$

where *H*_*i*_ is the immigration habitat and *H*_*j*_ is the selected emigration habitat. SIV is a suitability index variable which represents the feature of the habitat. Equation (3) means that the SIV of the habitat *H*_*i*_ can be replaced by the SIV of the selected habitat *H*_*j*_.

Mutation operator is a probabilistic one that can modify solution features, which is like mutation in many other EAs [19]. The purpose of mutation is to increase diversity among the population. The pseudocode of the canonical BBO is described in Algorithm 1.

Extensive works have been analyzed and discussed since BBO was proposed. With respect to different migration models corresponding to nature migration phenomenon, Ma [20] proposed six different migration models, among which sinusoidal migration curves perform the best. Additionally, some efficient migration operators and mutation operators have also been proposed to improve the performance of original BBO. Ma and Simon [5] proposed BBO with blended operator to solve constrained optimization problems. Guo et al. [7] further proposed the uniform version of extended migration operator (UEMO) to enlarge the space for offspring, thus avoiding local optimum to some extent. Zhang et al. [2] merged a differential mutation operator and a sharing operator into BBO's migration operator to balance the global and local search ability. Mi et al. [21] combined differential evolution mutation operators with simulated binary crosser of genetic algorithms. Apart from the above, some useful strategies borrowed from EAs have been applied to BBO. Gong et al. [22] combined differential evolution and BBO for numerical optimization. Zhang et al. [6] proposed a novel hybrid algorithm based on BBO and grey wolf optimizer to make full use of the two algorithms' search ability. Khademi et al. [23] took advantages of the feature-sharing capability of invasive weed optimization to enhance the performance of BBO. Lohokare et al. [24] accelerated BBO by adopting neighborhood search. To enhance the population diversity in BBO, opposition-based learning [25] and chaos strategy [25] have been introduced. Some theoretical studies of BBO can be found in [7, 26, 27].

Due to the simplicity and efficiency, BBO has been widely adopted in many engineering and science tasks. Bhattacharya and Chattopadhyay [28] solved both convex and nonconvex economic load dispatch problems of thermal plants with the assistance of BBO. Rahmati and Zandieh [29] developed an improved BBO to deal with flexible job shop scheduling problem. Niknamfar et al. [10] took advantage of BBO to handle a new hub-and-center transportation network problem. For further interest, readers can refer to some comprehensive reviews of BBO in [30, 31].

BBO in general performs well for most low-dimensional optimization problems; notwithstanding, its performance deteriorates rapidly when it comes to the high-dimensional problems. Unlike other optimization algorithms [17, 32, 33], few works on BBO aimed to scale up its performance. To the best of our knowledge, Guo et al. [7] made the first attempt to test their improved BBO with UEMO on large-scale optimization problems. However, UEMO does not outperform or cannot be even compared to the state-of-the-art large-scale algorithms. UEMO is the first attempt to handle LSOPs, but not yet scalable for LSOPs. With the advent of big data era, more and more optimization problems tend to involve thousands or even millions of decision variables. The scalable ability of EAs is crucial to deal with modern optimization problems. Therefore, in this work, we intend to scale up the performance of BBO.

### 2.2. Large-Scale Optimization

Large-scale optimization refers to the optimization problems with large numbers of decision variables. Although there is no formal definition of LSOPs, it is typically referred to the optimization problems in the high-dimensional space where conventional algorithms [17] suffer from the “curse of dimensionality” and fail to locate the optimum. Three reasons account for the failure: (1) with an increase of the decision variables, the corresponding search space will exponentially increase, which makes it difficult to optimize searching in such large space; (2) the characteristic of problem may be altered due to the increase of dimensionality; (3) evaluating LSOPs is time-consuming and sometimes unrealistic for real-world optimization problems which require to be solved in a reasonable time. Over the last decade, plenty of works have been proposed to copy with LSOPs. Basically, they can be divided into two categories: decomposition methods and nondecomposition methods.

#### 2.2.1. Decomposition Algorithms

Decomposition methods adopt the strategy of divide and conquer. It contains two steps, namely, decomposition and optimization. In the decomposition stage, a high-dimensional problem is decomposed into several low-dimensional subproblems which are easier to handle. In the optimization stage, each subproblem is evolved independently using one or several EAs. The final solution is a concatenation of representatives from each of the subproblem. Three crucial issues should be considered in this procedure, i.e., the decomposition accuracy, selection of optimizer, and computing resource allocation to the subcomponents.

The purpose of decomposition is to divide the interacting variables into a subcomponent, such that the global optimum can be obtained by evolving each low-dimensional subproblem independently. Early decomposition methods [17, 34, 35] does not explore variable interactions, thus failing to handle nonseparable problems. Recently, many research works have started to address this issue by implicitly or explicitly detecting the variable interactions. Sun et al. [36] proposed a statistical variable interdependence learning (SL) scheme based on nonmonotonic detection to explore variable interdependence. Omidvar et al. [37] proposed a differential grouping (DG) method based on nonlinear detection. To enhance the accuracy and efficiency of decomposition, some improved methods were proposed, such as extended DG (XDG) [38], DG2 [39], and recursive DG (RDG) [18].

Potter and De Jong [40] initially applied DC framework to improve the performance of GA. Since then, many metaheuristic algorithms, e.g., differential evolution [17], particle swarm optimization [34], and artificial bee colony [41], have demonstrated their superiorities in solving the LSOPs in the context of DC. Nevertheless, few works have focused on the scalability of some new yet efficient EAs, while in our study, we specifically scale up BBO to deal with LSOPs.

In the original DC framework, each subgroup is evolved in a round-robin fashion with equal computational budget allocated. It has been reported that the contribution of each subgroup to the global fitness of the individuals was in fact varied [42]. Omidvar et al. [42] proposed a contribution-based cooperative coevolution that selects the subgroup to be evolved according to their contributions to the global fitness. The contribution was calculated accumulatively, which can be greatly favored from the components with a good initial contribution. It cannot respond to the timely change of objective value in particular in the late phase of evolution. Therefore, Omidvar et al. [43] mended the contribution calculation formula later. Yang et al. [44] instead proposed to discard the stagnant components if detected so that the limited computing resource can be saved. Nevertheless, they might also remove the components that could be temporal stagnant. Different from above studies in serial computing environment, Jia et al. [45] proposed an adaptive resource allocation scheme in the distributed computing environment. Compared to other issues in the DC framework, computing resource allocation of subgroups has been paid less attention, however, which is closely related to practical application.

#### 2.2.2. Nondecomposition Algorithms

In addition to the CC, another research line to address the LSOPs is to improve the performance of traditional algorithms. Representative techniques include efficient initialization methods [46]; special operators for sampling and mutations [47, 48]; and hybrid algorithms [16] to accumulate strengths of different algorithms. To reduce the computation cost, surrogate model [49–51], and parallel computing [52, 53] have also been investigated to solve LSOPs.

Overall, it is meaningful to scale up the performance of BBO with the strategy of cooperative coevolution to deal with LSOPs in the big data era. Although DC has been embedded into canonical BBO, i.e., CBBO, it was only tested on functions of 30 dimensions [54]. The performance of CBBO on high-dimensional problems (larger than 100 dimensions) is still unknown. Hence, we propose a selective migration operator to balance the ability of exploration and exploitation; the DC framework is utilized as well where we introduce a more efficient strategy to allocate the limited computational budget.

## 3. Proposed Approach

### 3.1. Selective Migration Operator

A Heuristic Migration Operator (HMO) was proposed in reference [7]. Assuming that *H*_*j*_(SIV) is selected to immigrate from *H*_*i*_(SIV), if the fitness of *H*_*j*_(SIV) is better than that of *H*_*i*_(SIV), then *H*_*j*_(SIV) will share good information with *H*_*i*_(SIV) by migration. Otherwise, the migration will not happen. The heuristic migration operator can be represented as follows:

(4) $$\begin{array}{c} H_{i}\left(\text{SIV}\right)⟵H_{i}\left(\text{SIV}\right)+\alpha \left(H_{j}\left(\text{SIV}\right)-H_{i}\left(\text{SIV}\right)\right),\;f_{j}\leq f_{i}, \end{array}$$

where *α* ∈ [0, 1], *f* is the fitness value (we consider the minimization problem in our paper, unless otherwise specified). What's more, they extend the value of *α* ∈ [−0.25, 1.25] to enlarge the search area, which is called Uniform version of Extended Migration Operator (UEMO). In HMO and UEMO, the good emigrated individual intends to share promising information with the poor one, while the poor emigrated individual will not influence the good one. However, the current good individual will not be evolved in this generation, which degrades the exploitation ability. What's more, the global optimum is more likely to be located around these good individuals. Therefore, we design a Selective Migration Operator (SMO) to enhance the exploitation ability.

**Figure 2:**
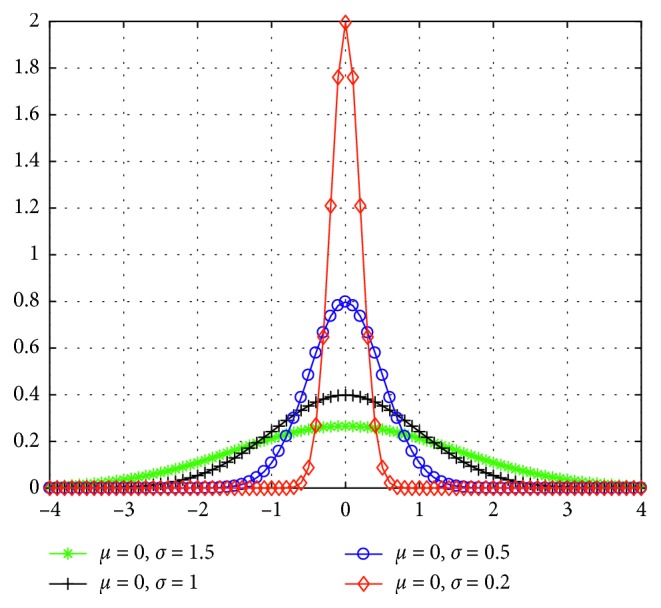
Normal distribution with various standard deviations.

To accelerate the convergence of the local search with better accuracy, we propose a normal distributed migration operator. The normal distribution curves with various standard deviations are shown in Figure 2. Since we focus on local search, smaller variations are preferred. Inspired by the HMO, we propose a Selective Migration Operator (SMO) to balance the exploration and exploitation. The selective migration operator can be represented as follows:

(5) $$\begin{array}{c} H_{i}\left(SIV\right)⟵H_{i}\left(SIV\right)+\beta \left(H_{j}\left(SIV\right)-H_{i}\left(SIV\right)\right),\;f_{j}\leq f_{i}, \end{array}$$

(6) $$\begin{array}{c} H_{i}\left(SIV\right)⟵H_{i}\left(SIV\right)+\gamma \left(H_{j}\left(SIV\right)-H_{i}\left(SIV\right)\right),\;f_{j}>f_{i}, \end{array}$$

where *β* is a variable close to 1, and *γ* is a normal distributed random number with smaller variations. In SMO, the poor immigrated individual will learn more useful information from good emigrated one, while the good immigrated individual will exploit its neighborhood area. The pseudocodes of SMO are given in Algorithm 2. Since the individuals in BBO are mutated towards random direction through mutation operator which may destroy good individuals, the mutation operator was removed. We use the selective migration operator to replace the original migration operator and name the corresponding algorithm selective biogeography-based optimization (SBBO).

### 3.2. Resource Allocation Based on Contribution

Since cooperative coevolution scheme is efficient for high-dimensional problems, we adopt DC framework for LSOPs in our paper. As we discussed above, it is unwise to assign equal computational budget to each subgroup due to the imbalanced contribution of them to the global fitness value. To address this issue, a contribution-based resource allocation scheme needs to be considered, which yields the essential question about how to measure each subgroup's contribution to the overall fitness value. The previous contribution calculation methods either focus too much on the initial good solutions [42] or brutally abandon the stagnant subgroups [44]. We instead calculate the contribution by the Relative Fitness Improvement (RFI). More specifically, the relative fitness improvement of subgroup *i* at generation *t* (generation refers to evolution of each subgroup) is defined as

(7) $$\begin{array}{c} {\text{RFI}}_{i}=\frac{f_{t-1}\left(H_{\text{best}}^{\prime }\right)-f_{t}\left(H_{\text{best}}\right)}{f_{t-1}\left(H_{\text{best}}^{\prime }\right)}, \end{array}$$

where *f*_*t*−1_(*H*_best_′) and *f*_*t*_(*H*_best_) refers to the best overall fitness value before and after subgroup *i* undergoes the evolution, respectively. In the first cycle (a cycle refers to a complete evolution of all subgroups), each subgroup is evolved by sequence. The RFI values of each subgroup is calculated according to equation (7) and stored in an archive. Then, the subgroup *i* with largest RFI value is selected to undergo evolution in the next generation. And the RFI value of the subgroup *i* is updated after evolution so that RFI is in a dynamic updated manner. The pseudocodes of resource allocation based on RFI are presented in Algorithm 3.

### 3.3. Proposed Method

As discussed above, to deal with the LSOPs in the context of DC, we propose to use SBBO as the base optimizer and allocate the computing resource to different subcomponents according to the RFI. Nevertheless, the computing resource will still be assigned to the subgroup of extremely small RFI value in the late phase of evolution. Thereby, the improvement of the overall best fitness value is not obvious. Other subgroups considered as stagnant ones before may be promising after several evolutions. Hence, to avoid wasting the computing resource on stagnant subgroup, an extra constraint is applied. If the RFI of subgroup *i* is smaller than a small value, it can be regarded as a temporal stagnant one and discarded from evolutionary cycle temporarily. If all the subgroups are considered as stagnant ones, each subgroup will be evolved equally, and the RFI will be updated completely. That is to say, the extra constraint added to the resource allocation strategy can further enhance the efficiency of computing budget. We name the SBBO, in the context of CC, with the resource allocation strategy after CC_SBBO_RA, although many different decomposition strategies have been proposed. Given decomposition accuracy and computational efficiency, we adopt RDG to divide the optimization problems in this paper [18]. Instead of detecting variables interactions in a pairwise manner, RDG can reduce the time complexity of decomposition by recursively examining the interaction between a selected decision variable and the remaining variables, such that more computational resource can be focused on the optimization stage. The pseudocodes of CC_SBBO_RA are shown in Algorithm 4.

## 4. Experiments

Experiments consist of three parts. First, some parameters need to be determined in CC_SBBO_RA. Hence, parameter sensitivity is analyzed in the first part. Second, the SBBO algorithm with DC framework is evaluated on CEC 2010 benchmark suite. BBO variants, SaNSDE [17], and CMA-ES [55] for LSOPs are compared with SBBO in terms of solution accuracy, since SaNSDE and CMA-ES are used in the context of CC, named as CC_SaNSDE CC-CMAES in the paper. In the third part, we provide the study of the contribution-based resource allocation in DC framework to show its effectiveness for LSOPs.

### 4.1. Benchmark Functions and Experimental Settings

The functions selected to evaluate the algorithm in our paper are CEC 2010 benchmark suite for LSGO [56]. Almost all LSO algorithms were evaluated on this benchmark suite. The CEC 2010 benchmark consists of 20 functions, listed in Table 1.

### 4.2. Parameter Sensitivity

**Figure 3:**
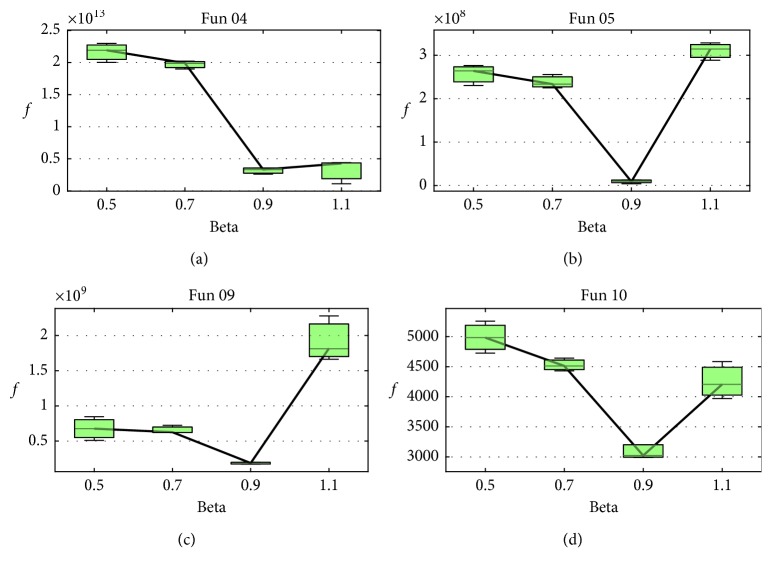
Change of the average fitness over the different *β* values (0.5, 0.7, 0.9, and 1.1) on *f*_4_, *f*_5_, *f*_9_, and *f*_10_.

In the proposed method, three parameters need to be determined before the experiment. In SBBO, *β*, a learning constant, determines how much information will be shared between the individuals. To investigate the constant *β*, we examine the change of fitness on both uni- and multimodal test problems with varying degrees of separability (*f*_4_, *f*_5_, *f*_9_, and *f*_10_ from Table 1). The fitness averaged over 25 independent runs as *β* increases is shown in Figure 3, from which we note that the fitness profiles on both uni- and multimodal problems with varying degrees of separability are a bit different. It is straightforward that *β* = 0.9 performs best. Through the fitness comparison, as we discussed above, only better individual's information can be emigrated to the evolved individual. As we all know, more good information sharing can result in faster convergence. Therefore, a large constant (close to 1) is preferred, which is confirmed in the experiments. When *β* = 0.5 or *β* = 0.7, only a relative small part of promising features can be shared, which degrades the information communication between individuals to some extent. When *β* is larger than 1, more uncertain information will be introduced to deteriorate the evolved individual. Hence, *β* = 0.9 is adopted here.

**Figure 4:**
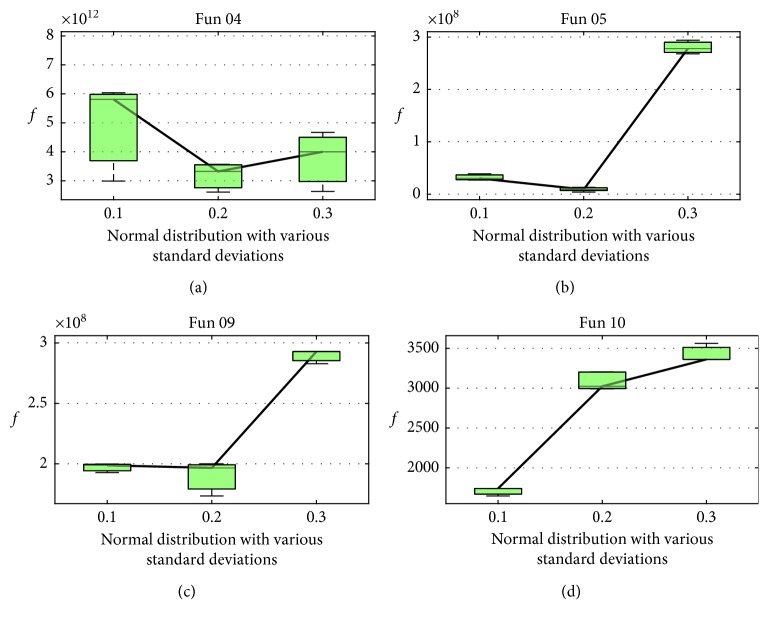
Change of the average fitness over *γ* with different variations (0.1, 0.2, and 0.3) on *f*_4_, *f*_5_, *f*_9_, and *f*_10_.

In BBO, *γ* is a normal distributed random number with smaller variations, which determines the local search ability. To investigate the appropriate variation, the same setting except the variation (0.1, 0.2, and 0.3), the change of fitness is shown in Figure 4. It is obvious that *γ* = norm (0 and 0.2) performs best except *f*_10_, which is a multimodal function. If the variation is 0.1, the local area is too small to search. While the variation is 0.3, the local search is too large so that it cannot be exploited enough. In this paper, *γ* = norm (0 and 0.2) is adopted.

**Figure 5:**
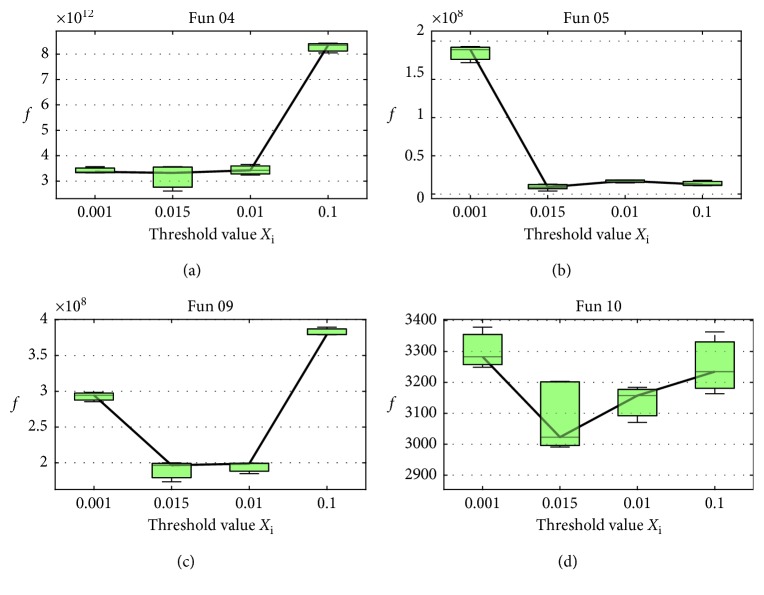
Change of the average fitness over *γ* with different threshold value on *f*_4_, *f*_5_, *f*_9_, and *f*_10_.

In the CC_SBBO_RA, the threshold value *ξ*, an extra constraint that determines which subgroup is in the temporal stagnation condition, needs to be explored in detail. As discussed above, RFI is used to measure each subgroup contribution, based on which the subgroup to be evolved is selected. That is to say, the smaller the RFI, the more likely the related subgroup to be stagnant. Since RFI is a relative value, we observe the change of fitness over different *ξ* values (0.1, 0.01, 0.015, and 0.001). When *ξ* is a large value (such as 0.1), as shown in Figure 5, the constraint will be too strict to determine stagnation. When *ξ* is too small, limited computing resource will be still assigned to stagnate subgroups. From the empirical experiment, *ξ* = 0.015 performs best, which is adopted in the paper.

### 4.3. Comparisons of BBO with Its Variants and Other Representative Algorithms

To the best of our knowledge, UEMO [7] was the first attempt to evaluate BBO variant's performance on LSOP benchmarks. UEMO adopted an extended migration operator to avoid the issue of shrinking the searching space due to blended migration operator. UEMO outperformed the original BBO w.r.t both best and average performance for LSOPs. As the best BBO variant for LSOPs, we compare our SBBO with it. Both algorithms are embedded into DC framework with the strategy of cooperative coevolution, every algorithm is called CC_Algorithm. The decomposition method adopted in our paper is RDG [18], which is the most accurate and efficient method so far. The total fitness evaluations (FEs) is 3e6 both for decomposition and optimization.

The best, mean, standard deviation values are presented in Table 2. CC_SBBO significantly outperforms CC_BBO on all benchmark problems. Furthermore, CC_SBBO, compared with CC_UEMO, achieves best solution quality on 17 benchmark functions and is competitive for the rest 3 functions. CC_SBBO's efficiency is attributed to the fact that the selective migration operator keeps the good exploration ability and focuses more on exploitation compared to the other migration operators.

SaNSDE [17], as a base optimizer, is widely used to solve LSOPs due to its efficiency, which adopts the strategy of neighborhood search and adaptation [57]. As an efficient and most used EA for LSOPs, CC_SaNSDE is compared with CC_SBBO, as shown in Table 2. CC_SBBO performs better than CC_SaNSDE on 5 benchmark functions, especially for fully separable functions. CC_SBBO can compete with CC_SaNSDE on function 6, 11, 14, 15, and 19. The good performance of CC_SBBO attributes to the proposed selective migration operator which increases its global search diversity and local search ability. In addition, migrated individuals and immigrated individuals of SBBO are selected according to the migration curve with a certain probability rather than random selection, which improves its performance to some degree. CC_SaNSDE performs better than CC_SBBO on the other 10 functions due to its varied neighborhood search operators and parameter adaptation. From the statistical results, CC_SBBO cannot beat CC_SaNSDE completely but it still has some advantages over CC_SaNSDE in some aspects as we mentioned before. Although both CC_SaNSDE and CC_SBBO perform worse than CC_CMAES on most functions, SaNSDE is still widely used as a base optimizer to deal with LSOPs due to its fast convergence. Analogue to SaNSDE, CC_SBBO provides us an alternative algorithm to deal with LSOPs, especially for some fully separable problems.

As an efficient algorithm for LSOPs, covariance matrix adaptation evolution strategy (CMA-ES) possesses a specific sampling strategy which samples offspring through a multivariate Gaussian distribution [58]. Also, this distribution is updated according to the offspring. From Table 2, CC_CMAES achieves best results on 13 functions due to its sampling strategy. The distribution estimated from the population can represent the correlation between decision variables. Thus, it is natural that CC_CMAES performs best on most partial separable and nonseparable functions, as indicated in [58]. Moreover, CC_CMAES can achieve good performance dealing with functions of rotation characteristic, and most test functions used in the paper possess the rotation characteristic. However, the performance of CC_CMAES deteriorates when it deals with fully separable and multimodal functions, such as function 2 and 3. Since there is no correlation between decision variables, the advantage of its sampling strategy declines to some extent. Moreover, CC_CMAES is more prone to getting stuck in local optimum when dealing with large-scale multimodal problems with no correlation between decision variables. We cannot ignore that some fully separable and multimodal problems do exist in the real world. In that cases, CC_SBBO can perform better than CC_CMAES according to Table 2. It is worth noting that, as pointed in [59], the initial candidate solution *x* ∈ *ℝ*^*n*^ and the initial global step size *σ* ∈ *ℝ*_+_ of CMA-ES must be chosen problem dependent, also, the optimum should presumably be within the cube *x* ± 2*σ*(1,…,1)^T^. That is to say, the parameters of CMA-ES need elaborate adjustment for different problems, while SBBO and SaNSDE are random initialized avoiding complex parameter tuning and are not limited to the region of the optimum. Furthermore, it is of promising potential to improve the performance of both SBBO and SaNSDE to cope with rotated functions by taking advantage of the characteristic of CMA-ES.

### 4.4. Efficiency of Resource Allocation

Contribution-based cooperative coevolution was first proposed to deal with imbalanced large-scale problems [42]. Each group is measured by the accumulated contribution, which shows preference for the good initial groups. The calculated contribution for each group *i* at cycle *t* can be expressed as follows:

(8) $$\begin{array}{c} \Delta f_{t}^{i}=\Delta f_{t-1}^{i}+f_{t-1}\left(H_{\text{best}}^{\prime }\right)-f_{t}\left(H_{\text{best}}\right), \end{array}$$

where *f*_*t*−1_(*H*_best_′) and *f*_*t*_(*H*_best_) refer to the best overall fitness value before and after subgroup *i* undergoes the evolution, respectively, and Δ*f*_*t*−1_^*i*^ is the calculated contribution of group *i* at cycle *t*−1. In this paper, we combine the aforementioned contribution measurement method with SBBO in the context of CC as the comparison algorithm, named as CC_SBBO_CB, and take it in comparison.

To save computation resource, the subgroups are out of evolution if they are considered as stagnant ones [44]. If mean and standard deviation of individuals remain unchanged for several successive generations, this subgroup is regarded as stagnation. To weaken the importance of initial good groups, they calculated the contribution of each group *i* at cycle *t* can be expressed as follows:

(9) $$\begin{array}{c} \Delta f_{t}^{i}=\Delta f_{t-1}^{i}+\frac{\left|\;f_{t-1}\left(H_{\text{best}}^{\prime }\right)-f_{t}\left(H_{\text{best}}\right)\right|}{2}. \end{array}$$

we consider the framework of resource allocation in the context of CC, and name it CC_SBBO_FR.

**Figure 6:**
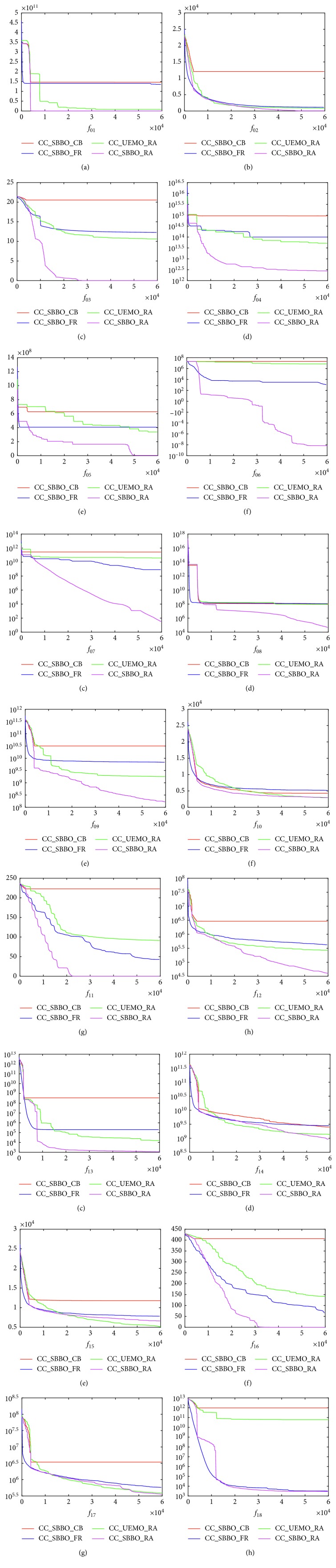
The evolution process of the best values on the CEC'2010 benchmark suite. The results were averaged over 25 runs. The vertical axis is the function value and the horizontal axis is the number of generations.

Our proposed computing resource allocation (RA) is considered both in CC_UEMO and CC_SBBO, called CC_UEMO_RA and CC_SBBO_RA correspondingly. The results are presented in Table 3, and the evolutionary process is shown in Figure 6. It can be seen from Figure 6 that our contribution-based computing resource allocation scheme can greatly enhance the convergence rate and the solution accuracy except for problems *f*_10_ and *f*_15_, which are multimodal functions and easy to be trapped in local optimum. It is obvious that CC_SBBO_CB can trap in local optimum easily due to its preference to good initial subgroups. Compared to CC_SBBO_CB and CC_SBBO_FR, our proposed resource allocation method can react quickly to the contribution change during evolution and hence decrease the computation budget on stagnant groups. Since *f*_19_ and *f*_20_ are totally nonseparate functions, we do not consider resource allocation between subgroups on these two scenarios. Therefore, CC_SBBO_RA performs best on separable and partial separable functions. To conclude, our proposed contribution-based resource allocation scheme performs efficiently for LSOPs.

## 5. Conclusion

In this paper, we propose a selective migration operator for BBO. The selective migration operator can enhance the exploitation ability as well as keep its good exploration ability compared with the original migration operator. When dealing with LSOPs, the cooperative coevolution framework is adopted in our paper. To address the imbalance contribution of each subgroup to the overall fitness value in the context of DC, a more efficient contribution-based resource allocation method is proposed. The relative performance improvement is utilized to measure the contribution as it reflects the recent improvements timely. Also, a threshold strategy, as an extra constraint, is adopted to measure whether the subgroup is stagnant. Computing resource will not be assigned to the stagnant subgroup in the cycle. The CEC'2010 large-scale benchmark functions were used to evaluate the performance of CC_SBBO_RA. From our experimental results, several conclusions can be drawn.

Firstly, BBO with selective migration operator can significantly improve the performance for LSOPs compared with other BBO variants, especially for those fully separable problems. Secondly, our proposed contribution-based resource allocation method can clearly enhance the EAs' performance when embedded into the DC framework.

In the future, we intend to improve the performance of BBO dealing with large-scale multimodal optimization problems. Also, it is interesting to explore an adaptive value for stagnation measurements with high accuracy.

## Figures and Tables

**Algorithm 1:**
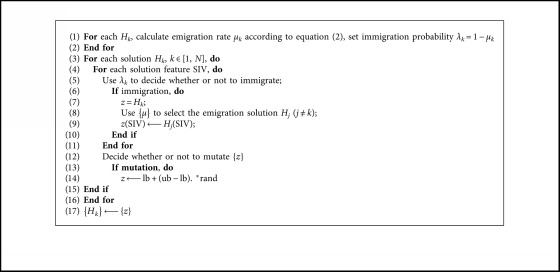
One generation of the canonical BBO algorithm, where *N* is the population size, *H*_*k*_ is the *k*th candidate solution, *H* is the entire solution, *H*_*k*_(SIV) is the feature of *H*_*k*_, *z* is a temporal solution, ub and lb are upper and lower bound of the search space, respectively.

**Algorithm 2:**
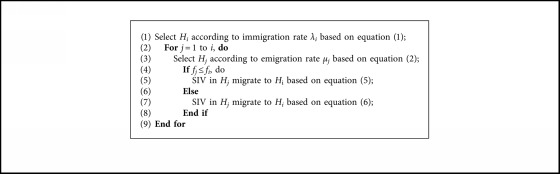
Pseudocodes of selective migration operator.

**Algorithm 3:**
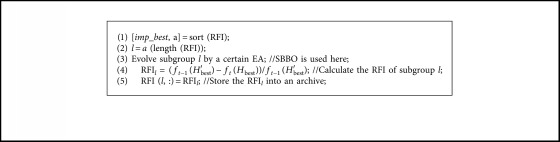
Pseudocodes of resource allocation based on RFI. Therein, RFI is the relative fitness improvement; *imp_best* is the largest RFI, *l* is the corresponding subgroup number.

**Algorithm 4:**
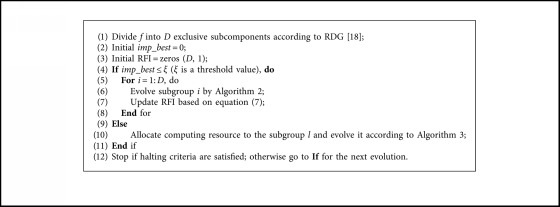
Pseudocodes of CC_SBBO_RA. Therein, *f* is an objective function; *D* is the number of subcomponents.

**Table 1:** The summary of functions in CEC 2010 benchmark suite.

Function name	Properties	Search range	Separability
F1: shifted elliptic function	Unimodal; shifted	[−100, 100]^*D*^	Fully separable
F2: shifted Rastrigin's function	Multimodal; shifted	[−5, 5]^*D*^	
F3: shifted Ackley's function	Multimodal; shifted	[−32, 32]^*D*^	

F4: single-group shifted 50-rotated elliptic function	Unimodal; shifted	[−100, 100]^*D*^	Single separable subcomponent
F5: single-group shifted 50-rotated Rastrigin's function	Multimodal; shifted	[−5, 5]^*D*^	
F6: single-group shifted 50-rotated Ackley's function	Multimodal; shifted	[−32, 32]^*D*^	
F7: single-group shifted 50-dimensional Schwefel's	Unimodal; shifted	[−100, 100]^*D*^	
F8: single-group shifted 50-dimensional Rosenbrock's	Multimodal; shifted	[−100, 100]^*D*^	

F9: 10-group shifted 50-rotated elliptic function	Unimodal; shifted	[−100, 100]^*D*^	*D*/2*m* separable subcomponents
F10: 10-group shifted 50-rotated Rastrigin function	Multimodal; shifted	[−5, 5]^*D*^	
F11: 10-group shifted 50-rotated Ackley function	Multimodal; shifted	[−32, 32]^*D*^	
F12: 10-group shifted 50-dimensional Schwefel's	Unimodal; shifted	[−100, 100]^*D*^	
F13: 10-group shifted 50-dimensional Rosenbrock's	Multimodal; shifted	[−100, 100]^*D*^	

F14: 20-group shifted 50-rotated elliptic function	Unimodal; shifted	[−100, 100]^*D*^	*D*/*m* separable subcomponents
F15: 20-group shifted 50-rotated Rastrigin's function	Multimodal; shifted	[−5, 5]^*D*^	
F16: 20-group shifted 50-rotated Ackley function	Multimodal; shifted	[−32, 32]^*D*^	
F17: 20-group shifted 50-rotated Schwefel's function	Unimodal; shifted	[−100, 100]^*D*^	
F18: 20-group shifted 50-rotated Rosenbrock's function	Multimodal; shifted	[−100, 100]^*D*^	

F19: shifted Schwefel's function 1.2	Unimodal; shifted	[−100, 100]^*D*^	Fully nonseparable
F20: shifted Rosenbrock's function	Multimodal; shifted	[−100, 100]^*D*^	

*Note. m* is the group size, and *D* is the dimension. In the CEC'2010 benchmark suite, *m* = 50, *D* = 1000.

**Table 2:** The results of the CC_BBO, CC_UEMO, CC_SBBO, and CC_SaNSDE algorithms on the CEC'2010 benchmark problems.

Function	Stats	CC_BBO	CC_UEMO	CC_SBBO	CC_SaNSDE	CC_CMAES
*f*_1_	Best	1.99*e* + 10↑	7.94*e* + 09↑	3.40*e* + 06	**8.42e** − **02**↓	1.31*e* + 05↓
	Mean	2.22*e* + 10	1.17*e* + 10	1.40*e* + 09	**2.07e** **+** **00**	2.84*e* + 05
	Std	1.60*e* + 09	1.01*e* + 10	1.13*e* + 09	**6.76e** **+** **00**	2.28*e* + 04

*f*_2_	Best	3.98*e* + 03↑	5.40*e* + 02‖	**4.62e** **+** **02**	4.12*e* + 03↑	2.81*e* + 03↑
	Mean	4.02*e* + 03	7.96*e* + 02	**1.07e** **+** **03**	4.41*e* + 03	4.43*e* + 03
	Std	6.43*e* + 01	1.88*e* + 02	**2.06e** **+** **02**	1.68*e* + 02	1.77*e* + 02

*f*_3_	Best	1.52*e* + 01↑	9.71*e* + 00‖	**2.42e** **+** **00**	1.64*e* + 01↑	8.66*e* + 00↑
	Mean	1.54*e* + 01	1.04*e* + 01	**1.07e** **+** **01**	1.66*e* + 01	1.06*e* + 00
	Std	1.31*e* − 01	8.01*e* − 01	**1.21e** **+** **00**	3.05*e* − 01	3.49*e* − 01

*f*_4_	Best	4.11*e* + 14↑	3.99*e* + 13↑	1.25*e* + 13	1.08*e* + 12↓	**8.45e** **+** **05**↓
	Mean	5.68*e* + 14	4.89*e* + 13	5.15*e* + 13	2.74*e* + 12	**1.01e** **+** **06**
	Std	1.19*e* + 14	8.56*e* + 12	2.96*e* + 13	3.19*e* + 12	**9.37e** **+** **04**

*f*_5_	Best	4.93*e* + 08↑	2.13*e* + 08↑	5.26*e* + 07	1.16*e* + 08↑	6.81*e* + 07‖
	Mean	5.12*e* + 08	2.84*e* + 08	1.88*e* + 08	1.28*e* + 08	9.52*e* + 07
	Std	1.15*e* + 07	4.99*e* + 07	7.20*e* + 07	1.92*e* + 07	2.23*e* + 07

*f*_6_	Best	1.63*e* + 07↑	2.66*e* + 06↑	1.59*e* + 01	1.73*e* + 01‖	**8.64e** − **01↓**
	Mean	1.65*e* + 07	7.62*e* + 06	9.19*e* + 01	1.83*e* + 01	**9.17e** − **01**
	Std	1.62*e* + 05	3.41*e* + 06	2.94*e* + 00	5.70*e* + 01	**4.23e** − **01**

*f*_7_	Best	9.27*e* + 10↑	1.33*e* + 10‖	8.92*e* + 09	2.07*e* + 01↓	**6.84e** − **19↓**
	Mean	1.02*e* + 11	2.07*e* + 10	2.44*e* + 10	2.16*e* + 01	**7.41e** − **19**
	Std	8.00*e* + 09	1.01*e* + 10	8.72*e* + 09	7.57*e* + 00	**8.35e** − **20**

*f*_8_	Best	1.72*e* + 15↑	4.09*e* + 12↑	2.06*e* + 08	3.14*e* + 05↓	**1.21e** − **17↓**
	Mean	2.34*e* + 15	1.61*e* + 15	5.95*e* + 14	5.59*e* + 05	**7.97e** **+** **05**
	Std	6.99*e* + 14	3.26*e* + 15	1.31*e* + 15	2.97*e* + 05	**1.63e** **+** **06**

*f*_9_	Best	5.90*e* + 09↑	1.13*e* + 09↑	2.04*e* + 08	4.28*e* + 07↓	**4.23e** **+** **06**↓
	Mean	6.26*e* + 09	1.54*e* + 09	1.11*e* + 09	4.70*e* + 07	**4.82e** **+** **06**
	Std	2.20*e* + 08	3.31*e* + 08	1.58*e* + 09	5.22*e* + 06	**5.25e** **+** **05**

*f*_10_	Best	7.03*e* + 03↑	3.12*e* + 03↑	2.48*e* + 03	4.26*e* + 03↑	2.64*e* + 03‖
	Mean	7.07*e* + 03	3.41*e* + 03	3.08*e* + 03	4.33*e* + 03	2.88*e* + 03
	Std	4.90*e* + 01	1.82*e* + 02	3.97*e* + 02	1.39*e* + 02	1.29*e* + 02

*f*_11_	Best	1.82*e* + 02↑	6.58*e* + 01↑	2.18*e* + 01	2.34*e* + 01‖	**1.49e** − **12**↓
	Mean	1.84*e* + 02	7.93*e* + 01	6.50*e* + 01	5.96*e* + 01	**3.58e** − **02**
	Std	9.94*e* − 01	1.03*e* + 01	2.12*e* + 01	2.75*e* + 01	**1.79e** − **01**

*f*_12_	Best	1.24*e* + 06↑	2.49*e* + 05↑	1.49*e* + 04	1.25*e* + 03↓	**3.12e** − **22↓**
	Mean	1.28*e* + 06	2.89*e* + 05	2.19*e* + 04	1.53*e* + 03	**4.23e** − **22**
	Std	2.32*e* + 04	4.15*e* + 04	1.11*e* + 03	4.66*e* + 02	**8.39e** − **23**

*f*_13_	Best	2.97*e* + 10↑	1.29*e* + 10↑	1.98*e* + 08	6.59*e* + 02↓	**3.21e** **+** **00↓**
	Mean	3.19*e* + 10	1.55*e* + 10	8.50*e* + 09	7.41*e* + 02	**5.90e** **+** **00**
	Std	2.19*e* + 09	1.91*e* + 09	7.23*e* + 09	2.57*e* + 02	**4.01e** **+** **00**

*f*_14_	Best	1.13*e* + 10↑	1.12*e* + 09↑	3.62*e* + 08	3.88*e* + 08‖	**3.17e** − **20↓**
	Mean	1.17*e* + 10	1.43*e* + 09	8.11*e* + 08	3.97*e* + 08	**3.91e** − **20**
	Std	2.73*e* + 08	2.79*e* + 08	5.62*e* + 08	2.31*e*+07	**2.12e** − **21**

*f*_15_	Best	1.01*e* + 04↑	4.45*e* + 03‖	4.41*e* + 03	5.78*e* + 03‖	1.91*e* + 03‖
	Mean	1.02*e* + 04	4.82*e* + 03	5.13*e* + 03	5.84*e* + 03	1.95*e* + 03
	Std	9.09*e* + 01	3.35*e* + 02	4.37*e* + 02	1.01*e* + 02	1.11*e* + 02

*f*_16_	Best	3.31*e* + 02↑	1.26*e* + 02↑	4.79*e* + 01	**2.56e** − **13**↓	8.24*e* − 13↓
	Mean	3.33*e* + 02	1.46*e* + 02	9.00*e* + 01	**2.67e** − **13**	8.44*e* − 13
	Std	1.33*e* + 00	1.80*e* + 01	1.49*e* + 01	**9.81e** − **15**	2.10*e* − 14

*f*_17_	Best	2.04*e* + 06↑	3.27*e* + 05↑	3.13*e* + 04	4.01*e* + 04↑	**6.72e** − **24**↓
	Mean	2.14*e* + 06	3.57*e* + 05	4.27*e* + 04	4.08*e* + 04	**6.91e** − **24**
	Std	8.06*e* + 04	1.99*e* + 04	3.64*e* + 03	2.56*e* + 03	**2.06e** − **25**

*f*_18_	Best	5.85*e* + 10↑	2.01*e* + 10‖	3.51*e* + 08	1.01*e* + 03↓	**1.46e** **+** **01**↓
	Mean	6.19*e* + 10	3.88*e* + 10	3.42*e* + 10	1.19*e* + 03	**1.50e** **+** **01**
	Std	1.95*e* + 09	1.70*e* + 10	1.48*e* + 10	1.69*e* + 02	**7.20e** **+** **00**

*f*_19_	Best	3.96*e* + 07↑	6.26*e* + 06↑	1.29*e* + 06	1.71*e* + 06‖	**5.31e** **+** **03↓**
	Mean	4.51*e* + 07	9.14*e* + 06	1.77*e* + 06	1.73*e* + 06	**5.47e** **+** **03**
	Std	7.12*e* + 06	3.61*e* + 06	5.50*e* + 05	7.52*e* + 04	**7.08e** **+** **02**

*f*_20_	Best	3.74*e* + 12↑	2.56*e* + 11↑	2.23*e* + 11	3.87*e* + 03↓	**8.47e** **+** **02↓**
	Mean	9.98*e* + 12	9.86*e* + 11	3.54*e* + 11	4.09*e* + 03	**8.27e** **+** **02**
	Std	4.46*e* + 11	4.94*e* + 11	1.25*e* + 11	3.29*e* + 03	**6.35e** **+** **01**

*Note.* The notation “↑/‖/↓” represents that CC_SBBO generated statistically “better/equally-well/worse” solution than the other algorithms. The best performances are highlighted bold.

**Table 3:** The results of the CC_SBBO_CB, CC_SBBO_FR, CC_UEMO_RA, and CC_SBBO_RA algorithms on the CEC'2010 benchmark problems.

Function	Stats	CC_SBBO_CB	CC_SBBO_FR	CC_UEMO_RA	CC_SBBO_RA
*f*_1_	Best	1.29*e* + 11↑	1.35*e* + 11↑	1.34*e* + 07↑	**0.00e** **+** **00**
	Mean	1.37*e* + 11	1.45*e* + 11	4.26*e* + 07	**6.14e** − **26**
	Std	1.04*e* + 10	1.08*e* + 10	2.35*e* + 07	**1.08e** − **25**

*f*_2_	Best	5.24*e* + 03↑	1.13*e* + 03↑	2.34*e* + 02↑	**4.37e** **+** **01**
	Mean	5.28*e* + 03	1.19*e* + 03	5.65*e* + 02	**6.24e** **+** **01**
	Std	4.97*e* + 01	4.16*e* + 01	4.41*e* + 02	**2.40e** **+** **01**

*f*_3_	Best	2.05*e* + 01↑	1.22*e* + 01↑	3.21*e* − 01↑	**1.17e** − **12**
	Mean	2.05*e* + 01	1.26*e* + 01	5.67*e* − 01	**1.79e** − **11**
	Std	2.74*e* − 02	2.05*e* − 01	4.82*e* − 01	**4.00e** − **11**

*f*_4_	Best	9.35*e* + 14↑	1.00*e* + 14↑	8.53*e* + 12↑	**7.99e** **+** **08**
	Mean	9.52*e* + 14	1.23*e* + 14	9.62*e* + 13	**9.17e** **+** **08**
	Std	1.07*e* + 13	2.42*e* + 13	8.64*e* + 12	**2.37e** **+** **08**

*f*_5_	Best	6.12*e* + 08↑	4.07*e* + 08↑	7.22*e* + 07↑	**3.98e** **+** **06**
	Mean	6.40*e* + 08	4.24*e* + 08	8.01*e* + 07	**4.21e** **+** **06**
	Std	7.46*e* + 07	1.80*e* + 07	5.26*e* + 07	**3.60e** **+** **06**

*f*_6_	Best	1.98*e* + 07↑	1.08*e* + 03↑	2.18*e* + 06↑	**7.10e** − **09**
	Mean	2.00*e* + 07	3.85*e* + 03	3.54*e* + 06	**8.90e** − **09**
	Std	3.69*e* − 01	2.39*e* + 03	2.43*e* + 06	**3.82e** − **09**

*f*_7_	Best	2.66*e* + 11↑	8.15*e* + 08↑	7.23*e* + 09↑	**1.23e** **+** **01**
	Mean	3.72*e* + 11	8.69*e* + 08	8.92*e* + 09	**2.18e** **+** **01**
	Std	3.86*e* + 02	4.44*e* + 07	7.18*e* + 09	**1.13e** **+** **01**

*f*_8_	Best	2.21*e* + 08↑	1.11*e* + 08↑	9.62*e* + 07↑	**4.82e** **+** **04**
	Mean	3.12*e* + 08	1.93*e* + 08	1.57*e* + 08	**5.33e** **+** **04**
	Std	6.34*e* + 07	6.28*e* + 07	8.29*e* + 07	**1.02e** **+** **04**

*f*_9_	Best	3.45*e* + 10↑	6.90*e* + 09↑	2.50*e* + 08‖	**1.73e** **+** **08**
	Mean	4.36*e* + 10	8.20*e* + 09	2.82*e* + 08	**1.77e** **+** **08**
	Std	1.65*e* + 09	9.71*e* + 08	7.82*e* + 06	**5.22e** **+** **06**

*f*_10_	Best	4.29*e* + 03↑	5.18*e* + 03↑	**2.31e** **+** **03‖**	2.99*e* + 03**‖**
	Mean	5.03*e* + 03	5.28*e* + 03	**2.41e** **+** **03**	3.00*e* + 03
	Std	3.09*e* + 02	7.76*e* + 01	**1.43e** **+** **02**	1.19*e* + 01

*f*_11_	Best	2.22*e* + 02↑	4.29*e* + 01↑	9.10*e* + 01↑	**8.52e** − **14**
	Mean	2.24*e* + 02	5.44*e* + 01	9.43*e* + 01	**9.87e** − **14**
	Std	1.34*e* + 00	1.33*e* + 01	3.42*e* + 00	**1.06e** − **14**

*f*_12_	Best	2.05*e* + 06↑	4.21*e* + 05↑	7.18*e* + 04↑	**4.04e** **+** **04**
	Mean	2.06*e* + 06	4.53*e* + 05	7.59*e* + 04	**4.47e** **+** **04**
	Std	2.63*e* + 04	2.88*e* + 04	4.25*e* + 03	**4.26e** **+** **03**

*f*_13_	Best	2.31*e* + 08↑	2.02*e* + 05↑	1.54*e* + 04↑	**1.11e** **+** **03**
	Mean	2.46*e* + 08	2.18*e* + 05	1.76*e* + 04	**1.60e** **+** **03**
	Std	1.16*e* + 07	1.03*e* + 04	1.84*e* + 02	**1.13e** **+** **02**

*f*_14_	Best	2.50*e* + 09↑	2.82*e* + 09↑	2.03*e* + 09↑	**9.20e** **+** **08**
	Mean	2.76*e* + 09	2.98*e* + 09	2.75*e* + 09	**9.97e** **+** **08**
	Std	2.69*e* + 08	1.04*e* + 08	2.47*e* + 08	**7.49e** **+** **07**

*f*_15_	Best	1.00*e* + 04↑	7.83*e* + 03‖	**5.29e** **+** **03‖**	6.09*e* + 03
	Mean	1.01*e* + 04	7.95*e* + 03	**5.81e** **+** **03**	6.24*e* + 03
	Std	1.01*e* + 02	1.22*e* + 02	**1.64e** **+** **02**	1.25*e* + 02

*f*_16_	Best	3.85*e* + 02↑	6.51*e* + 01↑	9.24*e* + 01↑	**9.05e** − **10**
	Mean	3.86*e* + 02	8.47*e* + 01	9.94*e* + 01	**1.18e** − **09**
	Std	1.92*e* + 00	1.63*e* + 01	5.48*e* + 01	**9.80e** − **10**

*f*_17_	Best	2.09*e* + 06↑	5.77*e* + 05‖	9.64*e* + 05↑	**2.13e** **+** **05**
	Mean	2.19*e* + 06	5.91*e* + 05	1.02*e* + 06	**3.12e** **+** **05**
	Std	7.14*e* + 04	1.60*e* + 04	3.28*e* + 06	**2.14e** **+** **05**

*f*_18_	Best	4.32*e* + 08↑	3.20*e* + 03‖	5.52*e* + 07↑	**2.75e** **+** **03**
	Mean	4.76*e* + 08	4.81*e* + 03	5.64*e* + 07	**2.96e** **+** **03**
	Std	3.91*e* + 07	1.07*e* + 03	1.25*e* + 07	**2.32e** **+** **03**

*Note.* The notation “↑/‖/↓” represents that CC_SBBO_RA generated statistically “better/equally-well/worse” solution than the other algorithms. The best performances are highlighted bold.

## Data Availability

The data used to support the findings of this study are available from the corresponding author upon request.

## References

[1] Simon D. (2008). Biogeography-based optimization. *IEEE Transactions on Evolutionary Computation*.

[2] Zhang X., Kang Q., Tu Q., Cheng J., Wang X. (2019). Efficient and merged biogeography-based optimization algorithm for global optimization problems. *Soft Computing*.

[3] Ma H., Yang Z., You P., Fei M. (2017). Multi-objective biogeography-based optimization for dynamic economic emission load dispatch considering plug-in electric vehicles charging. *Energy*.

[4] Guo W., Wang L., Wu Q. (2016). Numerical comparisons of migration models for multi-objective biogeography-based optimization. *Information Sciences*.

[5] Ma H., Simon D. (2011). Blended biogeography-based optimization for constrained optimization. *Engineering Applications of Artificial Intelligence*.

[6] Zhang X., Kang Q., Cheng J., Wang X. (2018). A novel hybrid algorithm based on biogeography-based optimization and grey wolf optimizer. *Applied Soft Computing*.

[7] Guo W., Wang L., Si C., Zhang Y., Tian H., Hu J. (2017). Novel migration operators of biogeography-based optimization and Markov analysis. *Soft Computing*.

[8] Rifai A. P., Nguyen H.-T., Aoyama H., Dawal S. Z. M., Masruroh N. A. (2018). Non-dominated sorting biogeography-based optimization for bi-objective reentrant flexible manufacturing system scheduling. *Applied Soft Computing*.

[9] Yang G., Liu Y. (2017). Optimizing an equilibrium supply chain network design problem by an improved hybrid biogeography based optimization algorithm. *Applied Soft Computing*.

[10] Niknamfar A. H., Niaki S. T. A., Niaki S. A. A. (2017). Opposition-based learning for competitive hub location: a bi-objective biogeography-based optimization algorithm. *Knowledge-Based Systems*.

[11] Ma H., Simon D. (2017). *Evolutionary Computation with Biogeography-Based Optimization*.

[12] Yang Z., Sendhoff B., Tang K., Yao X. (2016). Target shape design optimization by evolving B-splines with cooperative coevolution. *Applied Soft Computing*.

[13] Teng H. F., Chen Y., Zeng W., Shi Y. J., Hu Q. H. (2010). A dual-system variable-grain cooperative coevolutionary algorithm: satellite-module layout design. *IEEE Transactions on Evolutionary Computation*.

[14] Weber M., Neri F., Tirronen V. (2011). Shuffle or update parallel differential evolution for large-scale optimization. *Soft Computing*.

[15] Molina D., Lozano M., Sánchez A. M., Herrera F. (2011). Memetic algorithms based on local search chains for large scale continuous optimisation problems: MA-SSW-Chains. *Soft Computing*.

[16] Wang Y., Li B., Weise T. (2010). Estimation of distribution and differential evolution cooperation for large scale economic load dispatch optimization of power systems. *Information Sciences*.

[17] Yang Z., Tang K., Yao X. (2008). Large scale evolutionary optimization using cooperative coevolution. *Information Sciences*.

[18] Sun Y., Kirley M., Halgamuge S. K. (2018). A recursive decomposition method for large scale continuous optimization. *IEEE Transactions on Evolutionary Computation*.

[19] Whitley D. (1994). A genetic algorithm tutorial. *Statistics and Computing*.

[20] Ma H. (2010). An analysis of the equilibrium of migration models for biogeography-based optimization. *Information Sciences*.

[21] Mi Z., Xu Y., Yu Y., Zhao T., Zhao B., Liu L. (2015). Hybrid biogeography based optimization for constrained numerical and engineering optimization. *Mathematical Problems in Engineering*.

[22] Gong W., Cai Z., Ling C. X. (2010). DE/BBO: a hybrid differential evolution with biogeography-based optimization for global numerical optimization. *Soft Computing*.

[23] Khademi G., Mohammadi H., Simon D. (2017). Hybrid invasive weed/biogeography-based optimization. *Engineering Applications of Artificial Intelligence*.

[24] Lohokare M. R., Pattnaik S. S., Panigrahi B. K., Das S. (2013). Accelerated biogeography-based optimization with neighborhood search for optimization. *Applied Soft Computing*.

[25] Ergezer M., Simon D., Du D. Oppositional biogeography-based optimization.

[26] Simon D., Ergezer M., Dawei Du D., Rarick R. (2011). Markov models for biogeography-based optimization. *IEEE Transactions on Systems, Man, and Cybernetics, Part B (Cybernetics)*.

[27] Ma H., Simon D., Fei M. (2016). Statistical mechanics approximation of biogeography-based optimization. *Evolutionary Computation*.

[28] Bhattacharya A., Chattopadhyay P. K. (2010). Biogeography-based optimization for different economic load dispatch problems. *IEEE Transactions on Power Systems*.

[29] Rahmati S. H. A., Zandieh M. (2012). A new biogeography-based optimization (BBO) algorithm for the flexible job shop scheduling problem. *International Journal of Advanced Manufacturing Technology*.

[30] Guo W., Chen M., Wang L., Mao Y., Wu Q. (2017). A survey of biogeography-based optimization. *Neural Computing and Applications*.

[31] Ma H., Simon D., Siarry P., Yang Z., Fei M. (2017). Biogeography-based optimization: a 10-year review. *IEEE Transactions on Emerging Topics in Computational Intelligence*.

[32] Akay B., Karaboga D. (2012). Artificial bee colony algorithm for large-scale problems and engineering design optimization. *Journal of Intelligent Manufacturing*.

[33] Dong W., Chen T., Tino P., Yao X. (2013). Scaling up estimation of distribution algorithms for continuous optimization. *IEEE Transactions on Evolutionary Computation*.

[34] Li X., Yao X. (2012). Cooperatively coevolving particle swarms for large scale optimization. *IEEE Transactions on Evolutionary Computation*.

[35] Omidvar M. N., Li X., Yang Z., Yao X. Cooperative co-evolution for large scale optimization through more frequent random grouping.

[36] Sun L., Yoshida S., Cheng X., Liang Y. (2012). A cooperative particle swarm optimizer with statistical variable interdependence learning. *Information Sciences*.

[37] Omidvar M. N., Li X., Mei Y., Yao X. (2014). Cooperative co-evolution with differential grouping for large scale optimization. *IEEE Transactions on Evolutionary Computation*.

[38] Sun Y., Kirley M., Halgamuge S. K. Extended differential grouping for large scale global optimization with direct and indirect variable interactions.

[39] Omidvar M. N., Yang M., Mei Y., Li X., Yao X. (2017). DG2: a faster and more accurate differential grouping for large-scale black-box optimization. *IEEE Transactions on Evolutionary Computation*.

[40] Potter M. A., De Jong K. A. A cooperative coevolutionary approach to function optimization.

[41] Ren Y., Wu Y. (2013). An efficient algorithm for high-dimensional function optimization. *Soft Computing*.

[42] Omidvar M. N., Li X., Yao X. Smart use of computational resources based on contribution for cooperative co-evolutionary algorithms.

[43] Omidvar M. N., Kazimipour B., Li X., Yao X. CBCC3-a contribution-based cooperative co-evolutionary algorithm with improved exploration/exploitation balance.

[44] Yang M., Omidvar M. N., Li C. (2017). Efficient resource allocation in cooperative co-evolution for large-scale global optimization. *IEEE Transactions on Evolutionary Computation*.

[45] Jia Y. H., Chen W. N., Gu T. (2018). Distributed cooperative co-evolution with adaptive computing resource allocation for large scale optimization. *IEEE Transactions on Evolutionary Computation*.

[46] Segredo E., Paechter B., Segura C., González-Vila C. I. (2018). On the comparison of initialisation strategies in differential evolution for large scale optimisation. *Optimization Letters*.

[47] Yang Z., Tang K., Yao X. (2011). Scalability of generalized adaptive differential evolution for large-scale continuous optimization. *Soft Computing*.

[48] Tuo S., Zhang J., Yuan X., Yong L. (2018). A new differential evolution algorithm for solving multimodal optimization problems with high dimensionality. *Soft Computing*.

[49] Regis R. G. (2014). Evolutionary programming for high-dimensional constrained expensive black-box optimization using radial basis functions. *IEEE Transactions on Evolutionary Computation*.

[50] Li E., Wang H., Ye F. (2016). Two-level multi-surrogate assisted optimization method for high dimensional nonlinear problems. *Applied Soft Computing*.

[51] Sun C., Jin Y., Cheng R., Ding J., Zeng J. (2017). Surrogate-assisted cooperative swarm optimization of high-dimensional expensive problems. *IEEE Transactions on Evolutionary Computation*.

[52] Wang H., Rahnamayan S., Wu Z. (2013). Parallel differential evolution with self-adapting control parameters and generalized opposition-based learning for solving high-dimensional optimization problems. *Journal of Parallel and Distributed Computing*.

[53] Cano A., García-Martínez C. 100 million dimensions large-scale global optimization using distributed GPU computing.

[54] Zheng X.-W., Lu D.-J., Wang X.-G., Liu H. (2015). A cooperative coevolutionary biogeography-based optimizer. *Applied Intelligence*.

[55] Mei Y., Omidvar M. N., Li X., Yao X. (2016). A competitive divide-and-conquer algorithm for unconstrained large-scale black-box optimization. *ACM Transactions on Mathematical Software*.

[56] Tang K., Yáo X., Suganthan P. N. (2007). *Benchmark Functions for the CEC’ 2008 Special Session and Competition on Large Scale Global Optimization*.

[57] Yang Z., Tang K., Yao X. Self-adaptive differential evolution with neighborhood search.

[58] Hansen N. (2016). The CMA evolution strategy: a tutorial. https://arxiv.org/abs/1604.00772.

[59] Igel C., Hansen N., Roth S. (2007). Covariance matrix adaptation for multi-objective optimization. *Evolutionary Computation*.

